# The Use of Vigileo® Monitor in a Parturient With Severe Mitral Valve Stenosis and Severe Pulmonary Hypertension Undergoing Cesarean Section Under General Anesthesia: A Case Report and Literature Review

**DOI:** 10.7759/cureus.32284

**Published:** 2022-12-07

**Authors:** Panagiota Griva, Emmanouil Stamatakis, Giolanda Varvarousi, Anastasia Loukeri, Dimitrios Valsamidis

**Affiliations:** 1 Department of Anesthesiology, University General Hospital Attikon, Athens, GRC; 2 Department of Anesthesiology, Alexandra General Hospital of Athens, Athens, GRC

**Keywords:** intercultural mediators, vigileo monitor, pulmonary hypertension, cesarean section, mitral valve stenosis

## Abstract

We report a case of a 35-year-old pregnant female of Afghan origin who was admitted to the intensive care unit (ICU) because of pulmonary edema development when she was in the 30th week of gestation. During the bedside examination, the transthoracic echocardiogram (TTE) revealed severe mitral valve stenosis and pulmonary hypertension. The patient went into treatment with metoprolol for the control of tachycardia and furosemide for the prevention of fluid overload. During the 32nd week of gestation, the medical council decided on a cesarean section (CS) to be carried out under general anesthesia. The anesthesiologists decided to use the Vigileo monitor (Edwards Lifesciences, Irvine, CA, USA) as it is vitally important to approach fluid administration as fluid management is challenging concerning the obstetric patient. Vigileo monitoring is based on the invasive measurement of cardiac output (CO), cardiac index (CI), stroke volume (SV), and stroke volume variation (SVV). Fluid resuscitation based on hemodynamic parameters is a key component of patient care, especially in scenarios such as cardiovascular disease. This is the first case report where a Vigileo monitor was applied to a patient with severe mitral valve stenosis and severe pulmonary hypertension undergoing a cesarean section, which was accomplished without any complications. The patient was discharged from the hospital on the 12th postoperative day, hemodynamically stable.

Each immigrant woman, regardless of her financial, social, cultural, or any other situation, has the fundamental right to receive complete perinatal healthcare. Nevertheless, the most recent statistical data show that those women’s access to public healthcare is insufficient, leading to high rates of maternal mortality. The international medical community has to adapt to the new multicultural environment, and health services must be provided to this vulnerable population with the appropriate level of safety.

## Introduction

In recent years, maternal morbidity has shown a significant increase, with cardiovascular diseases being the most prevalent cause. It is estimated that 25% of maternal deaths in the USA are attributed to cardiovascular diseases [1], which happens also in high-income countries [1]. Mothers’ cardiovascular disease is a term that includes a wide range of diagnoses, such as congenital heart disorders, valvulopathies, heart failure, acute coronary syndromes, pulmonary hypertension, arrhythmias, and infectious endocarditis. For each one of those diagnoses, physicians should seriously take into consideration the severity of the disease, the comorbidities of the mother, and the obstetric risks [1]. Moreover, according to the most recent report on motherhood mortality on Mothers and Babies: Reducing Risk through Audits and Confidential Enquiries across the UK (MBRRACE-UK), heart diseases are reported to be the first cause of maternal mortality. The respective frequency is slightly higher than 1.6 per 100,000 gestations (Appendices) [2].

During gestation, the pregnant patient is subjected to significant anatomical and physiological changes in response to the normal development of the fetus so that the mother’s body will be able to adapt sufficiently to the labor process. The plasma volume gradually increases, with 50% of that increase being accomplished by the 34th week of gestation. However, the red blood cell (RBC) volume does not increase at the same rate, and as a result, pregnant patients normally present a decrease in their levels of hemoglobin (Hb) and hematocrit (Hct). Regarding the number of platelets (PLTs), it tends to decrease as the gestation progresses, but it remains within the normal range. Changes in the coagulation system lead to a hypercoagulable state in pregnancy, which increases the risk of thrombosis. Meanwhile, significant changes occur also in the mother’s cardiovascular system, since the early stages of gestation. Specifically, peripheral vasodilation is mediated by endothelial factors and leads to a reduction of the peripheral resistance of up to 25%-30%, with a compensatory rise of the cardiac output of up to 40%. The latter is being carried out mainly via the increase of the heart rate (HR) and, to a lower degree, via the increasing stroke volume. At the same time, heart preload is diminished because of the pressure exerted from the distended uterus to the inferior vena cava. The lung capillary wedge pressure, as well as the central venous pressure (CVP), does not increase significantly during gestation, in contrast to the pulmonary vascular resistance, which decreases in proportion to the peripheral vascular resistance. Also, lung capillary wedge pressure does not decrease, but the plasma colloidal osmotic pressure is reduced by up to 10%-15%, which results in the vulnerability of pregnant women to pulmonary edema. The labor leads to an additional rise in the cardiac output, while the uterine contractions cause an autohemotransfusion of 300-500 mL of blood back into the woman’s circulation [3].

## Case presentation

Α 35-year-old, 60 kg, 165 cm female of Afghan origin with a history of three labors and four gestations presented to Mytilene General Hospital at the 30th week of gestation complaining about persistent cough and dyspnea. The record of her medical (individual and family) history was difficult because the woman was only speaking her native language, and there was no accompanying person thereby to help with the translation. The transthoracic echocardiogram (TTE) revealed mitral valve stenosis and pulmonary hypertension. The patient was diagnosed with pulmonary edema, and the physicians decided her transport to the Alexandra General Hospital of Athens for further investigation and treatment.

**Figure 1:**
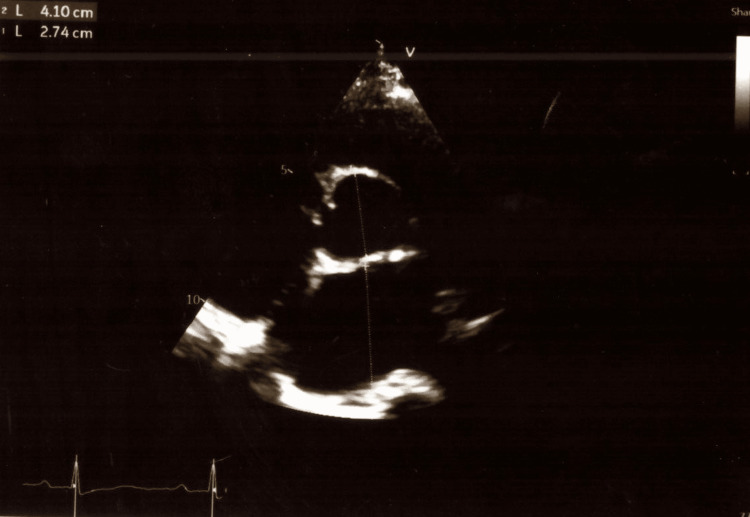
Two-dimensional echocardiogram from the patient with mitral valve stenosis

**Figure 2:**
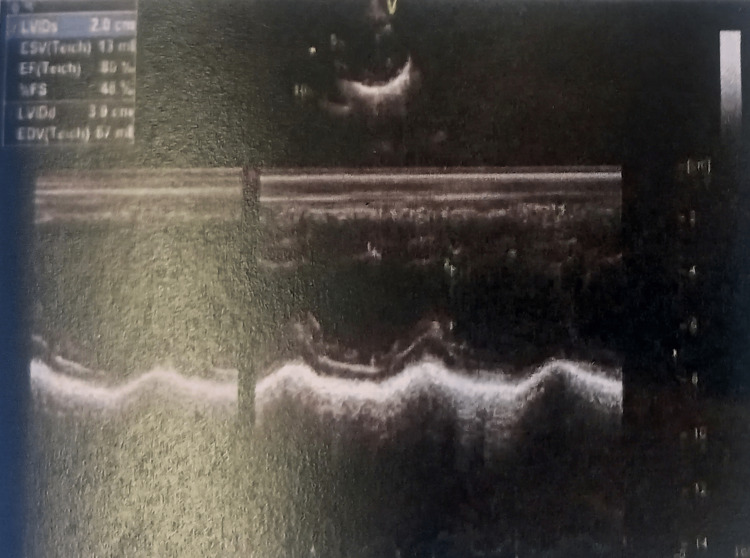
M-mode echocardiogram of the patient with mitral valve stenosis

**Figure 3:**
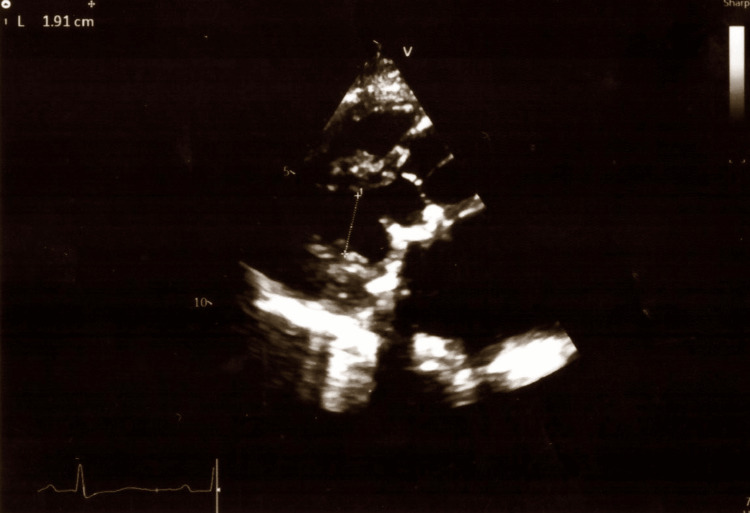
Parasternal long-axis echocardiogram of the patient with mitral valve stenosis

**Figure 4:**
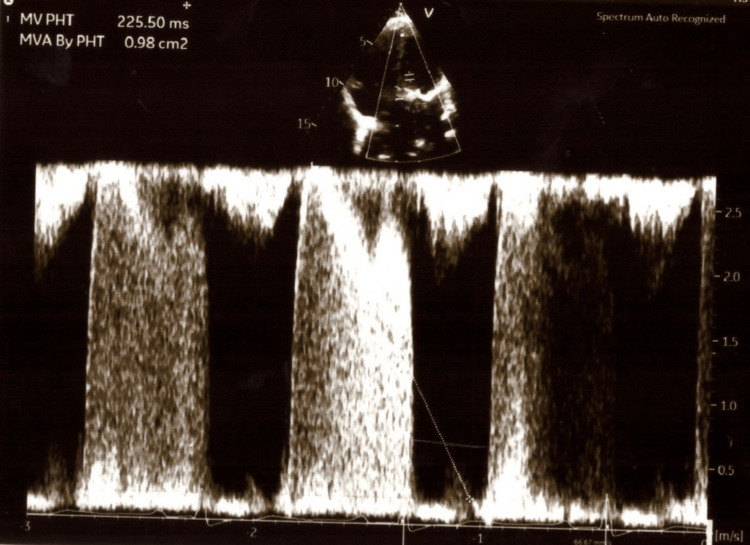
Transthoracic echocardiogram showing the mitral valve area

**Figure 5:**
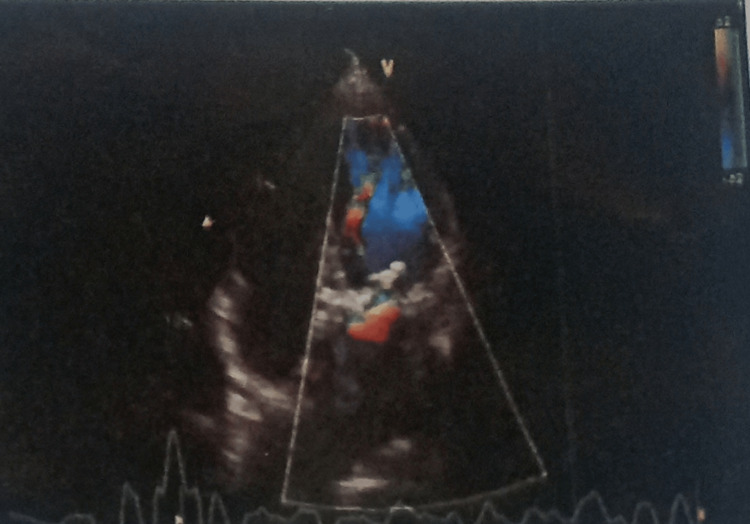
Color Doppler across the mitral valve in the patient with mitral valve stenosis

In the intensive care unit (ICU) of Alexandra General Hospital of Athens, a complete TTE examination was performed by the intensivists. Table 1 shows all her echocardiographic measurements. The ultrasonographic examination of her lungs showed pleural effusions in both lungs as well as the existence of b-lines in her pulmonary parenchyma. Images of the TTE examination are presented in Figures 1-5.

**Table 1:** Echocardiographic measurements PHT: pressure half-time, MVA: mitral valve area, VTI: velocity time integral, PASP: pulmonary artery systolic pressure, EF: ejection fraction, ΙVC: inferior vena cava

Measurements	Results
Mitral valve	Rheumatic attack with an arched morphology of the front cusp and fixation of the rear cusp
	Severe stenosis and moderate to severe insufficiency
	Maximum diastolic slope of blood pressure: 27 mmHg
	Moderate diastolic slope of blood pressure: 14 mmHg
	Anatomic outlet: 1.2 cm^2^
	PHT = 192 ms
	MVA from PHT = 1.1 cm^2^
Aortic valve	Trileaflet, without any opening restriction
	Low-grade failure, with ΑοVmax = 1.44 m/s
	VTI about 16 cm
	PHT = 539 ms
Tricuspid valve	Low-grade failure
	PASP = 66 mmHg
Pulmonary valve	Low-grade failure
Left ventricle	Moderate diffuse hypokinesia with an affected contractility
	EF = 50%
Left atrium	Dilated: 54 mm (normal range: 19-40 mm)
Right ventricle	Initiating dilatation
Right atrium	Within normal range
Ascending aorta	28 mm
Inferior vena cava	ΙVC = 16 mm with >50% respiratory variation

The patient was hospitalized for two weeks, and her medication included metoprolol 50 mg 1x3 for the control of her tachycardia and furosemide in proportion to her needs so that fluid overload would be avoided. Administration of betamethasone in two doses of 12 mg was very important due to fetal lung immaturity.

In view of her mitral valve stenosis and pulmonary hypertension, a cesarean section (CS) was scheduled by the medical council on the 32nd week of gestation, after daily monitoring of the fetal activity with cardiotocography with the assent of the neonatologists. Before surgery, a new TTE examination of the mother’s heart function was performed, which showed an increase in diastolic pressure gradient and a decrease in pulmonary artery systolic pressure (PASP) (Table 2).

**Table 2:** Echocardiographic measurements in the ICU ICU: intensive care unit, MV: mitral valve, PASP: pulmonary artery systolic pressure

	Admission to the ICU	24 hours before the cesarean section	24 hours after the cesarean section
Mean gradient MV (mmHg)	20	13	11
PASP (mmHg)	60	55	45

The patient was transferred to the operating room, where a six-lead electrocardiogram (ECG) and pulse oximetry (SpO2) were applied. A 20-gauge radial arterial cannula and a 7.5-French triple lumen right jugular vein catheter were inserted. Hemodynamic variables, including cardiac output (CO), cardiac index (CI), stroke volume (SV), stroke volume variation (SVV), stroke volume index (SVI), and invasive blood pressure (BP), were continuously recorded using Vigileo® monitor (Edwards Lifesciences, Irvine, CA, USA). Images of the Vigileo® monitor are presented in the figure below. Her hemodynamic parameters during CS are reported in Table 3, while intraoperative monitoring of arterial blood pressure, heart rate, and CVP is presented in Table 4.

**Table 3:** Hemodynamic parameters CO: cardiac output, CI: cardiac index, SVV: stroke volume variation, SV: stroke volume, SVI: stroke volume index

	Right after the delivery of the newborn	Right after the administration of oxytocin	20 minutes after intubation	Just before extubation
CO (L/minute)	5.1	5	5.3	6.8
CI (L/minute/m^2^)	3	2.9	3.1	4
SVV (%)	7	4	2	7
SV (mL/b)	55	60	65	67
SVI (mL/b/m^2^)	32	35	38	39

**Table 4:** Intraoperative monitoring of blood pressure, heart rate, and CVP CVP: central venous pressure

	Introduction in the operating room	Right after the neonate was delivered	Right after the administration of oxytocin	20 minutes after intubation	Just before extubation
Blood pressure (mmΗg)	101/63	104/70	98/57	111/67	115/71
Heart rate (bpm)	109	92	80	68	75
CVP (mmHg)	6	9	12	13	10

During her physical examination, she developed mild sinus tachycardia (109 bpm), and S1-S2 cardiac sounds were rhythmic, without any murmur. The rest of her physical examination did not show any pathological findings. For aspiration prophylaxis, the patient was administered metoclopramide 10 mg intravenous (IV) and cimetidine 200 mg IV. She was also administered cefuroxime 1.5 g IV for perioperative chemoprophylaxis and furosemide 20 mg IV for the prevention of fluid overload. Simultaneously with double preoxygenation with an anesthetic face mask (100%) and a nasal catheter at 5 L/minute, remifentanil 0.05 μg/kg/minute was administered by continuous IV infusion. When the expired oxygen percentage was 90%, etomidate 16 mg IV, succinylcholine 100 mg IV, and remifentanil 80 μg IV bolus were administered for the induction of anesthesia.

**Figure 6:**
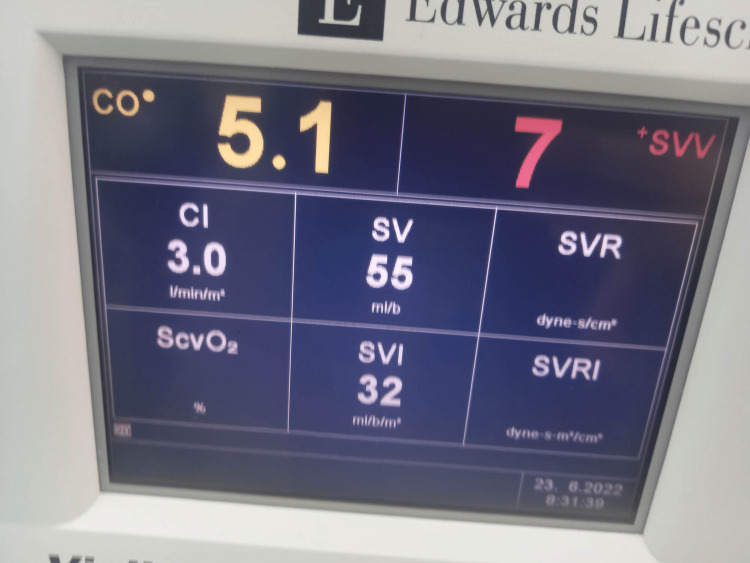
Vigileo® monitor right after the delivery of the newborn CO: cardiac output, CI: cardiac index, SV: stroke volume, SVV: stroke volume variation, SVI: stroke volume index

Soon after the induction of anesthesia and the application of mechanical positive pressure ventilation with tidal volume (TV) of 450 mL, respiratory rate (RR) of 12 breaths/minute, and positive end-expiratory pressure (PEEP) of 6 cm H2O and 50% FiO2, the patient remained hemodynamically stable (heart rate, 92 bpm; BP, 104/70 mmHg). The first hemodynamic parameters are presented in Figure 6.

Maintenance of anesthesia was performed by sevoflurane at 0.8% in an oxygen/air mix and continuous IV infusion of remifentanil at a rate of 0.05-0.1 μg/kg/minute.

The neonate was delivered within three minutes after induction, and the appearance, pulse, grimace, activity, and respiration (APGAR) score was 8 and 9 at the first and fifth minute of birth, respectively.

After delivery, the patient was administered three units of oxytocin bolus, followed by a slow infusion of another 17 units within 60 minutes. The vasodilation caused by oxytocin did not affect the patient hemodynamically. Morphine 10 mg IV and paracetamol 1 mg IV were also administrated after delivery. The arterial blood gases after delivery revealed the following data: pH, 7.38; PCO2, 39 mmHg; PO2, 170 mmHg; Hct, 34%; Hb, 10.5 g/dL; lactate acid (Lac), 1 mmol/ L; base excess (ΒΕ), -2 mmol/L; and HCO3, 23.1 mmol/L.

**Figure 7:**
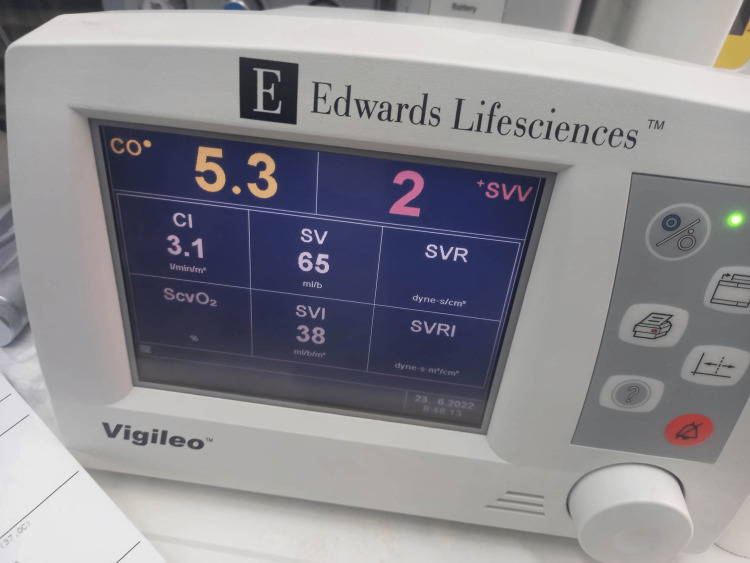
Vigileo® monitor 20 minutes after intubation CO: cardiac output, CI: cardiac index, SV: stroke volume, SVV: stroke volume variation, SVI: stroke volume index

**Figure 8:**
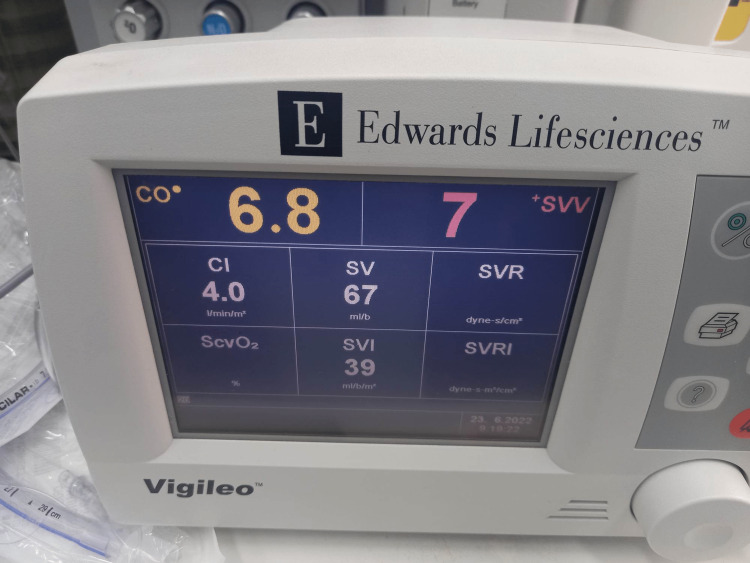
Vigileo® monitoring just before extubation CO: cardiac output, CI: cardiac index, SV: stroke volume, SVV: stroke volume variation, SVI: stroke volume index

Twenty minutes after the induction of anesthesia, SVV decreased from 7% to 2% (Figure 7), and central venous pressure (CVP) was 13 mmHg (Table 2). According to the above measurements, the patient was administered an additional dose of furosemide 20 mg IV to prevent fluid overload. The total duration of the surgical procedure was 45 minutes. The total amount of crystalloids administrated was 500 mL, and after furosemide administration, the urine output was 150 mL. The hemodynamic parameters just before the extubation are presented in Figure 8.

Soon after the extubation, the patient developed sinus tachycardia, which was successfully treated with the administration of esmolol 50 mg IV bolus, followed by a continuous infusion of 0.03 mg/kg/minute. The hemodynamically stable patient (BP, 115/67 mmHg; HR, 86 bpm; SpO2, 100% with a nasal catheter at 4 L/minute) was transferred to the ICU. Intravenous patient-controlled analgesia (PCA) with morphine was chosen for postoperative analgesia. Her pain was assessed using the visual analog scale (VAS) and was found to be as mild as VAS < 3.

On the first postoperative day, the patient was hemodynamically stable (Table 4). On the fifth postoperative day, the patient was discharged from the ICU and was transferred to the hospital’s gynecological ward, still hemodynamically stable in medication with metoprolol 5 mg 1x3 and furosemide 40 mg 1x1. During the 12th postoperative day, the patient was discharged from the hospital hemodynamically stable to the refugee camp.

## Discussion

This is the first case report where a Vigileo® monitor was applied to a patient with severe mitral valve stenosis and severe pulmonary hypertension undergoing cesarean section under general anesthesia. The catheterization of the pulmonary artery was considered too invasive, especially since the medical team was able to use the transesophageal ultrasound, after the intubation. Furthermore, in their article, McLean et al. reported that the cardiac output measurement performance of the Vigileo® program was comparable to that of the transesophageal Doppler in terms of reliability, with the only exception the cases of patients suffering from arrhythmia or severe aortic valve stenosis [4]. Further studies have also reported that the Vigileo® system’s reliability is comparable to that of the Swan-Ganz catheter, concerning the measurements of the cardiac output, peripheral resistances, and intravascular volume status [5]­­.

The successful fluid administration throughout the operation of the presented case study was assisted by the Vigileo® monitor. The contribution of the Vigileo monitor was crucial concerning the administration of diuretics. Without the image of the advanced hemodynamic parameters estimated by Vigileo®, the optimization of fluid status in this patient would be unattainable. An SVV value of <13% is a predictor of volume overload [6]. In our case, we could either eliminate the fluid uptake or administrate diuretics. In the first scenario, the fluid balance would be disturbed and fail to compensate for fluid losses during cesarean section.

As presented in Table 3, CO (normal range: 4-8 L/minute) and CI (normal range: 2.5-4.2 L/minute/m^2^) did not exceed the normal ranges at any stage of the surgery. Twenty minutes after intubation, the measurement of SVV was 2% (dropped from a 7% initial value). CVP was raised to 13 mmHg while SVV was reduced to 2%. This led to the administration of 20 mg furosemide, and it was decided that hereinafter, the receiving fluids would be restricted to 5 mL/kg/hour, meaning that the total amount of crystalloids administrated was 500 mL until the end of the operation.

In our regular practice, based on the mean arterial pressure, heart rate, and urinary output, we would not administrate a repeat dose of furosemide, and we would continue the fluid administration at a rate slightly higher than 5 mL/kg/hour. More aggressive fluid therapy and volume overload would have been associated with harmful consequences, including pulmonary edema. In conclusion, Vigileo was a useful tool for the fluid replacement of ongoing losses during cesarean section ensuring hemodynamic stability and avoiding volume overload. Stroke volume variation (SVV) is a reliable factor of fluid responsiveness [7,8], which can be used to guide fluid assessment in mechanically ventilated patients. Using SVV and Vigileo cardiac output monitor is associated with better intraoperative hemodynamic stability and avoidance of hazardous complications, especially considering cardiovascular diseases.

As illustrated in Table 2 and Table 3, after the above intervention, SVV was increased to 7% and CVP decreased to 10 mmHg. Since these parameters were reverted to their normal values, it can be deduced that we avoided fluid overload and prevented pulmonary edema. SVV is a significant hemodynamic parameter, with a critical value of 13%, which denotes that patients exceeding this value need immediate administration to replenish the fluid loss, whereas patients with lower SVV often have no response to fluid administration [6]. The patient should be kept normovolemic. Any possible fluid volume that she might need during the operation should be administered with caution since it might lead to pulmonary edema, which is more likely to occur after the labor. According to recent studies, the appropriate volume for a fluid challenge is either 250 mL bolus (crystalloid or colloid solutions) or 3 mL/kg of colloid solution at an infusion rate of 5-10 minutes [9]. Generally, the possibility of pulmonary edema in pregnant women with mitral valve stenosis is approximately 45%, as reported in the literature [10]. It is often the result of the decompression of the inferior vena cava combined with the autohemotransfusion (that happens due to uterine contractions), which leads to a preload increase. The suggested treatment involves the elevation of the head and the supply of 100% O2, while in extreme cases, even intubation might be necessary, accompanied by the application of mechanical ventilation with PEEP [11].

The type of anesthesia (either general or regional) depends on various factors, several of which are the severity of the disease, pulmonary hypertension, or coagulopathies. Generally, subarachnoid anesthesia is considered inappropriate in cases where severe mitral valve stenosis is detected [12].

In our case study, we decided that general anesthesia was more appropriate taking into consideration its advantages: avoidance of sudden drops in systemic vascular resistance (SVR), no need for volume preload or co-load, possible immediate treatment of acute atrial fibrillation and cardioversion, maintenance of adequate venous return, and definite securement of the airway in case of acute pulmonary edema. Moreover, general anesthesia has the advantage of preventing pain, hypoxemia, hypercarbia, and acidosis [12]. The aforementioned situations could possibly increase pulmonary vascular resistance [12]. Epidural anesthesia cannot offer less hypotension than general anesthesia. Moreover, we secured better cardiovascular monitoring with the Vigileo monitor as the literature supports the use of SVV only in patients who are 100% mechanically ventilated [6].

Regardless of the type of anesthesia, the anesthesiologist should avoid tachycardia. Tachycardia, alongside the pain during labor, increases the blood flow through the mitral valve, which causes a sudden increase in the pressure of the left atrium and leads to pulmonary edema [12]. With our anesthetic technique, we achieved the above hemodynamic goal as the heart rate decreased by 30% from the baseline (Table 4). Aiming to minimize tachycardia and hypertension during laryngoscopy, simultaneously with preoxygenation, remifentanil was initiated at an infusion rate of 0.05-0.1 μg/kg/minute. Remifentanil was chosen based on its sufficient analgesia levels and its rapid onset and short duration of action. Since it passes through the placenta and gets metabolized rapidly afterward, getting redistributed to both the mother and the fetus, remifentanil provides great intraoperative hemodynamic stability, as well as rapid recovery from anesthesia. It also prevents prolonged respiratory depression in the fetus [13]. The neonatologists were informed about the maternal administration of remifentanil to manage neonatal respiratory depression. It should also be noted that during the cesarean section, it is important to maintain a sufficient anesthesia depth to hinder any tachycardia or hypertension development. Taking this into consideration, sevoflurane in a minimum alveolar concentration (MAC) of 0.8 was utilized to maintain the desired anesthesia depth and avoid uterus atony. Also, it was decided that nitrous oxide should not be supplied, mainly due to its tendency to induce pulmonary hypertension.

A cardio-selective β1-blocker was also utilized for tachycardia treatment during the patient extubation stage. Specifically, esmolol 50 mg bolus IV was administered, and a continuous infusion at a rate of 0.3 mg/kg/minute was started. This decision was based on its rapid initial activation and short-duration properties.

It is important to mention that remifentanil provides insufficient postoperative analgesia due to the short duration of action. For postoperative analgesia, morphine 10 mg IV was administered right after the neonate was delivered. In cases of severe valvulopathy, the suggested treatment involves the administration of opioids, as they have only a slight suppressive action on the cardiovascular system while they provide an excellent level of analgesia. Further pharmacological agents that are reported to increase heart rate, for instance, ketamine, should be avoided. Intravenous patient-controlled analgesia (PCA) with morphine was chosen for postoperative analgesia as it is important to ensure a sufficient level of postoperative analgesia to avoid tachycardia.

More frequently, women with valvulopathies tend to develop cardiac comorbidities and obstetric complications during gestation and labor. The most frequent valvulopathy is mitral valve regurgitation, followed by mitral valve stenosis, tricuspid valve insufficiency, aortic valve insufficiency, aortic valve stenosis, and pulmonary valve stenosis. Mitral valve stenosis represents about 12% of all valvulopathies that have to be admitted to the hospital, while rheumatoid stenosis represents 85% of them [14].

Acute rheumatic fever is the main cause of mitral valve stenosis. Other causes include congenital valve stenosis and vegetations and calcifications of the cusps of the mitral valve. Acute rheumatic fever is attributed to group A *Streptococcus*, and its incidence is high in developing countries, possibly because of the fact that in those countries, access to healthcare services is limited and the provision of antibiotics is insufficient [15].

At the same time, it is estimated that 60% of untreated patients develop chronic stenosis of the mitral valve due to the final enlargement of the cusps of the mitral valve and the fusion of the papillary muscles. Epidemiologically, although both sexes are equally affected by acute rheumatic fever, more frequently, women tend to develop mitral valve stenosis (2:1) for unknown reasons [16].

In mitral valve stenosis that occurs as a result of acute rheumatic fever, the obstruction of the blood flow via the mitral valve that occurs due to the reduction of the width of the opening causes an increase in the systolic pressure of the left atrium and of the pulmonary arterial pressure [14].

Moderate and severe mitral valve stenosis are not well tolerated during gestation. The normal physiological changes that occur in pregnancy lead to tachycardia, which results in further reduction of the diastolic filling period. Pregnant women are at increased risk of heart failure, even those who are asymptomatic, especially during their second and third trimesters of gestation [17].

During prenatal screening tests, physicians should take a full medical history of the pregnant woman (arrhythmias, angina pectoris symptomatology, etc.), a full physical examination (murmurs, lower limbs edemas, etc.), and a full electrocardiographic and TTE examination [10]. Medical history of former cardiac complications or pulmonary hypertension is typically followed by higher rates of complications.

The aim of the treatment of mitral valve stenosis is the avoidance of medical conditions (anemia, thyroid disease, fever, infections, etc.) that increase heart rate and reduce the filling time of the left ventricle, thus resulting in a reduction in heart output. The cornerstone of that treatment lies in the administration of β-blockers, which reduce heart rate and increase the filling time of the left ventricle, with a subsequent increase in heart output. The most frequently used β-blockers are cardioselective β1-blockers, such as metoprolol and bisoprolol. Atenolol should be avoided in pregnant women because it is responsible for delayed fetal development [10]. Restrictions on fluids and dietary salt are also recommended. In the case of pulmonary edema, diuretics are the first-line treatment. However, the risk of hypovolemia is present, resulting in hypoperfusion of the umbilical vein and compensatory tachycardia. Anticoagulant treatment is indicated only in atrial fibrillation, the presence of a thrombus in the left atrium, or the patient’s history of a thromboembolic episode. It should also be administrated to women who have sinus rhythm with left atrial volume > 60 mL/m^2 ^[18].

Most pregnant women will respond to the above pharmacological treatment. However, a low percentage will still be categorized as stages III and IV according to the New York Heart Association (NYHA), or they will have a PASP of more than 50 mmHg. In these cases, a percutaneous mitral commissurotomy (PMC) will be needed, which is mainly carried out during the second trimester of gestation [18]. In the present case study, the patient was already in her third trimester of gestation, but she responded well to treatment, as dyspnea was improved and, as can be seen in Table 4, the pulmonary resistance fell dramatically. Furthermore, PMC is contraindicated in cases of moderate and severe mitral valve stenosis [11]. Data in Table 1 show that our patient had moderate to severe mitral valve insufficiency, so there was no indication for PMC. For the reasons above, PMC was not carried out before the labor. Rare complications of PMC have been reported: cardiac tamponade (1%-2%), thromboembolic event (1%), and death (<1%) for the mother and prematurity and mortality (one-third of the cases) for the fetus. No developmental delay has ever been reported [18].

It should also be considered that the presented case study needed special handling due to its social dimension. In 2011, Canada was the first country to publish guidelines for the medical treatment of immigrant women [19]. Each one of them, regardless of their state of health or financial and social situation, has the fundamental right to receive complete perinatal healthcare. However, recent statistics indicate that the access of those women to public healthcare is insufficient, with subsequent high rates of mortality [20]. Those rates are attributed to the poor health of those women, the ignorance of the physicians about the cultural ethics of their countries of origin (e.g., amputation of female genitals), the attitude of their husbands, and insufficient healthcare services in the host country. For those reasons, healthcare professionals should carefully examine the medical history of those women, which may be incomplete for various reasons, for instance, fear that it might endanger the woman’s stay in the host country, unavailability of medical records in the country of origin, and, most commonly, delay in the search for perinatal care [19]. Language barriers should not be an obstacle in the provision of the proper healthcare services to those women, and for that reason, the existence of intercultural mediators is crucial. Those mediators have to act impartially as translators to secure the unhampered transmission of information from the patient to the physicians. Their replacement by members of the patients’ families is considered to be risky because the language barrier still remains, and also, the objectivity of the transmitted information can be questioned.

In Greece, the special program “Intercultural Mediation” is implemented since 2009. The aim of that program is the familiarization of healthcare providers with multiculturalism so that the provided healthcare services will be of greater quality. That program, however, is applied only in several Greek hospitals. Subsequently, there is an imperative need for the publication of specific guidelines so that effective treatment of pregnant immigrant women can be applied. Since 2011, a 24-hour translating facilitation program via telephone has been running at the Alexandra General Hospital of Athens. Furthermore, professional translators of the most commonly spoken languages are also available (via physical appearance) during the morning working hours, opting to give immigrant patients easier access to medical care.

## Conclusions

The anesthesiologist, obstetrician, and cardiologist ought to collaborate closely, starting from the second trimester of pregnancy, and build a medical plan to handle mitral valve stenosis and secure a gestation without dangerous complications. It is certain that the healthcare services provided to each patient have to be individualized and any possible comorbidities should be taken into consideration. Regardless of the type of anesthesia, tachycardia and fluid overload must be avoided as those conditions have an impact on the hemodynamic stability of the patient. In our case study, we decided that general anesthesia was more appropriate taking into consideration its advantages. During the CS, we decided to use the Vigileo® monitor as it is vitally important to approach fluid administration as fluid management is challenging concerning the obstetric patient. Without the image of the advanced hemodynamic parameters estimated by Vigileo®, the optimization of fluid status in this patient would be unattainable. The total amount of crystalloids administrated was 500 mL. In addition, the contribution of the Vigileo® monitor was crucial concerning the administration of diuretics. Based on the abrupt decrease of SVV, she was administered furosemide 20 mg IV to prevent fluid overload. The total amount of furosemide administrated was 40 mg. Aiming to minimize tachycardia and hypertension during laryngoscopy, simultaneously with preoxygenation, remifentanil was initiated at an infusion rate of 0.1 μg/kg/minute. Esmolol, a cardioselective β1-blocker, was also utilized for tachycardia treatment during the patient extubation stage. Hence, we avoided the development of pulmonary edema, and the cesarean section was accomplished without any complications. The patient was discharged from the hospital on the 12th postoperative day, hemodynamically stable without any postoperative complications.

## Figures and Tables

**Figure 9:**
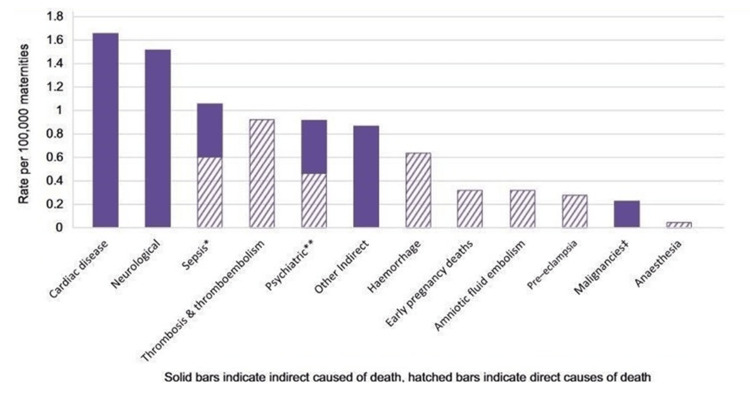
Causes of maternal mortality per 100,000 gestations Hatched bars show direct causes of death, and solid bars indicate indirect causes of death. *The rate for direct sepsis (genital tract sepsis and other pregnancy-related infections) is shown in hatched bars, and the rate for indirect sepsis (influenza, pneumonia, and others) is shown in solid bars. **The rate for suicides (direct) is shown in hatched bars, and the rate for indirect psychiatric causes (drugs/alcohol) is shown in solid bars. ‡Rate for indirect malignancies (breast/ovary/cervix). Source: Mothers and Babies: Reducing Risk through Audits and Confidential Enquiries across the UK (MBRRACE-UK) [2] This figure is reproduced under the permission of Professor Marian Knight, Fellowship of the Academy of Medical Sciences (FMedSci), MBRRACE-UK Maternal Programme Lead, Professor of Maternal and Child Population Health, Honorary Consultant in Public Health.

## Appendices

Figure 9 presents the causes of motherhood mortality per 100,00 gestations according to the most recent report of Mothers and Babies: Reducing Risk through Audits and Confidential Enquiries across the UK (MBRRACE-UK).
